# A Rare Case of *Sphingomonas paucimobilis* Meningitis in the Absence of Cerebrospinal Fluid Pleocytosis

**DOI:** 10.1177/2324709618756424

**Published:** 2018-01-31

**Authors:** Hassan Mehmood, Noman Khan, Saad Ullah, Asad Ullah, Asghar Marwat

**Affiliations:** 1 Temple University/Conemaugh Memorial Medical Center, Johnstown, PA, USA

**Keywords:** meningitis, *Sphingomonas paucimobilis*, neck rigidity, headache, meropenem

## Abstract

*Sphingomonas paucimobilis* is a nonfermentative gram-negative *bacillus* of low pathogenicity. The organism has been involved in causing a wide range of infections in community and hospital settings. Only 3 cases of meningitis caused by this organism have been reported so far. We report a rare case of *S paucimobilis* meningitis who presented with atypical symptoms. A 50-year-old female presented with headache, dizziness, chills, shakiness, and neck pain along with nuchal rigidity. On physical examination, severe neck rigidity along with decreased range of motion was noticed. Her cerebrospinal fluid showed gram-negative rods, and she was started on meropenem. The cerebrospinal fluid grew *S paucimobilis* sensitive to meropenem. She subsequently showed significant improvement and was discharged home on intravenous meropenem for 21 days and showed complete recovering in 5 weeks.

## Introduction

*Sphingomonas paucimobilis* is a nonfermentative gram-negative *bacillus* of low pathogenicity.^1-3^ The organism has been involved in causing a wide range of infections including septic arthritis, bacteremia, ventilator-associated pneumonia, and urinary tract infection both in the hospital setting and in the community.^1,4,5^ *S paucimobilis* can cause infection both in immunocompetent and immunocompromised hosts. Only 3 cases of meningitis caused by this organism have been reported so far.^6-8^ We report a rare case of *S paucimobilis* meningitis who presented with atypical symptoms.

## Case Report

A 50-year-old female with past medical history of rheumatoid arthritis came to our hospital with headache, dizziness, chills, shakiness, and neck pain along with nuchal rigidity. She had these symptoms going on for 3 to 4 days. Neck pain along with nuchal rigidity got worse the same day she presented to the hospital. On clinical examination, vitals included temperature of 36.6°C, blood pressure of 163/99 mm Hg, respiratory rate of 18 breaths per minute, and heart rate of 73 beats per minute. Severe neck rigidity with decreased range of motion was noticed. The rest of the clinical examination was unremarkable. Initial laboratory studies showed white blood cell count of 11 500/µL with 85% neutrophils and 13% lymphocytes, and sodium, creatinine, and hepatic panel was within normal limit. Computed tomography scan of neck was done along with magnetic resonance imaging of the brain, and both were unremarkable. Further workup included a lumbar puncture, which showed clear fluid with opening pressure of 10 mm/Hg, and cerebrospinal fluid (CSF) analysis revealed white blood cell count of 5/mm^3^ with 4% neutrophils, red blood cell count of 95/mm^3^, protein of 37 mg/dL, and glucose of 60 mg/dL. Gram stain of CSF showed gram-negative rods. Intravenous meropenem was initiated, and peripheral blood and CSF were sent for culture. Repeat lumbar puncture the next day was also unremarkable for any change in CSF analysis. Further blood work showed erythrocytes sedimentation rate of 32 mm/h, C-reactive protein of 0.4 mg/dL, and CSF was negative for herpes simplex virus. On hospital day 4, CSF grew *S paucimobilis* sensitive to meropenem. Her headache, dizziness, neck pain, and rigidity started showing improvement after 5 days and continued thereafter. She showed significant improvement after 10 days and was subsequently discharged to home with home health nurse to complete 21 days of intravenous meropenem. She was seen in the office 5 weeks after the initial presentation with complete return to her baseline and no more neck pain and rigidity.

## Discussion

*Sphingomonas paucimobilis* is an oxidase-positive, glucose nonfermenting, aerobic gram-negative *bacillus*.^1-3^ The bacterium was first identified in 1977 and originally named as *Pseudomonas paucimobalis*. In 1990, the bacterium was renamed *Sphingomonas Paucimobalis*.^4^ *S paucimobilis* is an opportunistic pathogen with relatively low virulence likely because of lack of typical gram-negative lipopolysaccharide cell membrane component as well as lack of endotoxin activity.^6^ *S paucimobilis* is widely distributed in nature. It can be found in soil, water, and in multiple hospital settings. It can be found in indwelling catheter, sterile intravenous fluid, implantation devices, and contaminated hospital environment such as ventilator, nebulizer, and dialysis devices.^4,9^ *S paucimobilis* was not considered to be a major pathogen in the past, but most recent data showed this idea needs to be reevaluated.^10^

In 31% to 52.7% of cases, infection is community acquired, and primary bacteremia is the source of infection in 44.8% of cases.^4,5^ *S paucimobilis* mostly cause infection in immunocompromised patient along with patients having multiple comorbidities including diabetes mellitus, liver diseases, end-stage renal disease, malignancy, and among patients having indwelling catheter and devices.^5^ Despite having prevalence in multiple settings, only 3 cases of *S paucimobilis* meningitis has been reported so far. Among those patients 2 were immunocompetent and 1 was immunocompromised.^6-8,11,12^

Acute bacterial meningitis mostly presents with fever, neck stiffness, and neurological symptoms. Classic triad can be present in some patient. Multiple studies showed missing of one or more symptoms in acute bacterial meningitis.^13-16^ However, virtually all patients have at least one of the findings of the classic triad of fever, neck stiffness, and neurological symptoms.^17,18^

The diagnosis of acute bacterial meningitis is mostly clinical along with CSF findings. In the right clinical setting headache, neck stiffness, and unexplained fever are highly suggestive of bacterial meningitis. The usual CSF findings are white blood cell count of 1000 to 5000/µL with the percentage of neutrophils usually greater than 80%, protein >200 mg/dL, and glucose <40 mg/dL. However, there have been reports shown in multiple studies where bacterial meningitis has no CSF abnormalities.^19,20^ The absence of classical CSF findings in our patient makes it a very clinical entity in the settings of positive CSF culture for *S paucimobilis*.^21^

*Sphingomonas paucimobilis* is usually susceptible to carbapenems, aminoglycosides, trimethoprim sulfamethoxazole, and piperacillin/tazobactam. Some studies showed resistance to penicillin and first-generation cephalosporin is because of the production of chromosomally encoded β-lactamase production.^1,22^ Third-generation cephalosporin and fluoroquinolones showed variable susceptibility.^1,23^ There is no definitive guidelines for antibiotic therapy for *S paucimobilis* and it should be done with individualized antibiotic therapy according to in vitro susceptibility profile of clinical specimen.^24^

## Conclusion

In conclusion, we are presenting a case of meningitis due to an unusual organism. Our case is extremely rare as there are only 3 cases of *S paucimobilis* meningitis reported so far. Our patient also had normal CSF findings and this type of presentation is extremely rare in adults with only 26 cases reported so far. We recommend raising awareness in the health care profession about *S paucimobilis* meningitis in the need of time. Detailed history and physical examination is key for the early diagnosis of meningitis. If there is high degree of suspicion of meningitis based on history and physical examination, empirically starting antibiotics can be extremely beneficial for the patient in terms of morbidity and mortality as normal CSF does not always rule out meningitis and clinicians must recognize that many exceptions exit. Treatment is mainly antibiotics along with supportive therapy and it is too early to determine the overall prognosis as very little data are available so far.
